# Laurel Attenuates Dexamethasone-Induced Skeletal Muscle Atrophy In Vitro and in a Rat Model

**DOI:** 10.3390/nu14102029

**Published:** 2022-05-12

**Authors:** Huijuan Jia, Takanori Yamashita, Xuguang Li, Hisanori Kato

**Affiliations:** Health Nutrition, Department of Applied Biological Chemistry, Graduate School of Agricultural and Life Sciences, The University of Tokyo, 1-1-1 Yayoi, Bunkyo-ku, Tokyo 113-8657, Japan; akakeiken@g.ecc.u-tokyo.ac.jp (H.J.); asuparapapupapu@gmail.com (T.Y.); lixuguang@g.ecc.u-tokyo.ac.jp (X.L.)

**Keywords:** laurel, skeletal muscle atrophy, proteolysis, MuRF1, C2C12 myotubes, rat

## Abstract

Prevention of muscle atrophy contributes to improved quality of life and life expectancy. In this study, we investigated the effects of laurel, selected from 34 spices and herbs, on dexamethasone (DEX)-induced skeletal muscle atrophy and deciphered the underlying mechanisms. Co-treatment of C2C12 myotubes with laurel for 12 h inhibited the DEX-induced expression of intracellular ubiquitin ligases—muscle atrophy F-box (atrogin-1/MAFbx) and muscle RING finger 1 (MuRF1)—and reduction in myotube diameter. Male Wistar rats were supplemented with 2% laurel for 17 days, with DEX-induced skeletal muscle atrophy occurring in the last 3 days. Laurel supplementation inhibited the mRNA expression of MuRF1, regulated DNA damage and development 1 (Redd1), and forkhead box class O 1 (Foxo1) in the muscles of rats. Mechanistically, we evaluated the effects of laurel on the cellular proteolysis machinery—namely, the ubiquitin/proteasome system and autophagy—and the mTOR signaling pathway, which regulates protein synthesis. These data indicated that the amelioration of DEX-induced skeletal muscle atrophy induced by laurel, is mainly mediated by the transcriptional inhibition of downstream factors of the ubiquitin-proteasome system. Thus, laurel may be a potential food ingredient that prevents muscle atrophy.

## 1. Introduction

Skeletal muscle atrophy decreases motor function, contributing to locomotive syndrome, and resulting in a vicious cycle of increased risk of falls, fractures, a bedridden condition, and further skeletal muscle atrophy [1]. The concept of locomotive syndrome was proposed by the Japan Orthopedic Association in 2007 and is defined as a high-risk condition that requires early treatment owing to impaired locomotive organs [2]. One of the main causes of locomotive syndrome is the deterioration of motor function with age, and sarcopenia is a well-known loss of skeletal muscle mass that occurs with age. With a rapidly increasing old age population worldwide, the number of patients suffering from sarcopenia and disuse-induced muscular dystrophy is expected to increase. This leads to overall worse outcomes, requiring hospitalization, and increasing medical expenses and bed occupancy rates [3].

Currently, there is still a lack of effective treatments for sarcopenia and skeletal muscle atrophy, partly owing to insufficient understanding of the pathological mechanisms that induce atrophy [4]. At present, the clinical therapy of muscle atrophy mainly includes non-steroidal anti-inflammatory drugs (NSAIDs) owing to the perception that clinical symptoms could be improved only by relieving pain, which suppresses arthritis symptoms. However, NSAID usage is presumed to be accompanied by adverse effects. Furthermore, machine learning has predicted that this drug class exerts several harmful drug–drug interactions [5,6].

Notably, resistance exercise is effective in promoting muscle mass recovery following muscle atrophy [7]. However, it is difficult for the elderly and bedridden patients to exercise on a daily basis to promote muscle growth. Therefore, fervent efforts have been made to devise strategies that limit the development of such chronic diseases; the strategies include lifestyle countermeasures, such as dietary modification [8,9]. Leucine, a branched-chain amino acid, has been used as a food-based intervention to combat skeletal muscle atrophy and is reported to activate the mechanistic target of rapamycin complex 1 (mTORC1) [10,11,12]. Similarly, vitamin E has beneficial effects on muscular atrophy, which are associated with the normalization of the mRNA expression of muscle atrophy F-box (atrogin-1/MAFbx) and muscle RING finger 1 (MuRF1) genes, rather than with its antioxidant effect [13,14]. In a previous study, we reported that genistein suppresses sciatic nerve resection-induced skeletal muscle atrophy in mice [15].

Spices and herbs are widely used in cooking and are reported to contain many bioactive substances, which exert several beneficial effects including anti-inflammatory, cognitive enhancement, and anti-cancerous properties [16,17]. It was previously reported that transient receptor potential vanilloid type 1 (TRPV1) promotes the process of muscle hypertrophy, especially induced by skeletal muscle overload and/or exercise [18]. The study also indicated that the administration of capsaicin, an agonist of TRPV1, suppressed muscular atrophy induced by hindlimb suspension and sciatic nerve resection. However, the beneficial effects of spices and herbs on skeletal muscle atrophy are insufficiently understood, and the underlying mechanisms remain uninvestigated. Therefore, the present study aimed to explore the effects of laurel (*Laurus nobilis*), which was selected from 34 types of spices and herbs, using dexamethasone (DEX)-stimulated mouse C2C12 myotubes and rat skeletal muscle-derived L6 cells, as well as a rat model of skeletal muscle atrophy.

## 2. Materials and Methods

### 2.1. Extraction of Spices

The spice and herb samples were purchased from a shopping market (Rakuten Group, Inc., Tokyo, Japan). The extraction was performed by incubating the spices (25 g) overnight in methanol (100 mL) in the dark at 23 °C ± 2 °C. The supernatant was spontaneously filtered through a filter paper (Toyo Roshi, No. 5B, Tokyo, Japan) to obtain the filtrate (i) and residue (ii). The residue (ii) was washed twice with 10 mL methanol and spontaneously filtered through No. 5B filter paper to obtain the filtrate (ii). The filtrates (i) and (ii) were mixed, and the solvent was distilled off using an evaporator (bath temperature 40 °C) to obtain the extract.

### 2.2. Cell Culture

Rat skeletal muscle-derived L6 myoblast cells and mouse skeletal muscle-derived C2C12 cells were obtained from the American Type Culture Collection (Rockville, MD, USA). These myoblasts were cultured in Dulbecco’s modified Eagle’s medium (DMEM, Sigma-Aldrich, Santa Clara, CA, USA) supplemented with 10% fetal bovine serum (FBS; Gibco, Australia), 100 units/mL penicillin, and 100 μg/mL streptomycin (Gibco) in humidified air at 37 °C with 5% CO_2_. To induce the differentiation of myoblasts into myotubes, L6 cells and C2C12 cells at 90% confluence were cultured in DMEM containing 2% FBS for 9 days and 2% horse serum for 5 days, respectively. After the induction of differentiation, cells were detached from the surface using trypsin and resuspended in DMEM.

### 2.3. Determination of Cell Cytotoxicity

The toxicity of laurel toward myotube cells was detected using the lactate dehydrogenase (LDH) assay (Wako Pure Chemical Industries, Osaka, Japan). Briefly, based on the aforementioned cell culture method, C2C12 cells were seeded in 24-well plates at a density of 2.5 × 10^4^ cells per well and cultured for 5 days in DMEM containing 2% horse serum, 100 units/mL penicillin, and 100 μg/mL streptomycin (Gibco). After the induction of differentiation, the cells were cultured in a serum-free medium for 12 h. The C2C12 cells were then incubated for 12 h with laurel at concentrations of 5–150 μg/mL. The medium was then aspirated, and collected in tubes, and the cells were collected with DMEM containing 1% tween-20. The assay was performed according to the manufacturer’s instructions, and absorbance (Abs) was measured at 550 nm using a spectrophotometer (Thermo Fisher Scientific, Waltham, MA, USA). The formula used to calculate cytotoxicity is as follows:

Cytotoxicity (%) = (Abs of medium)/(Abs of medium + Abs of cells) × 100

### 2.4. Measurement of Myotube Diameter

C2C12 myotubes were cultured in serum-free DMEM for 12 h. The cells were then pretreated with 10 nM DEX for 12 h followed by laurel treatment at 50 nM for 12 h. Images were taken using a microscope (Leica Microsystems GmbH, Wetzlar, Germany) at ×100 magnification, and diameters of myotubes were semi-quantitatively determined for 80 random fields of C2C12 cells using the Image J software (Media Cybernetics, Bethesda, MD, USA).

### 2.5. Animals and Experimental Design

Healthy male Wistar rats (8 weeks old) were purchased from CLEA Japan, Inc. (Tokyo, Japan) and acclimated for 6 days in an animal facility at a temperature of 22 ± 1 °C and humidity of 60% ± 5%, with a reversed light/dark cycle (12:12 h). The rats were provided water and normal feed ad libitum. The normal feed was a powdered feed based on AIN-93G; the composition of each feed is shown in Supplementary Table S1. All experimental procedures and animal health care were reviewed and approved by the Animal Experiment Committee of the University of Tokyo and were conducted according to the University of Tokyo Experimental Procedures.

In total, 21 rats were randomly divided into three groups (*n* = 7/group) as follows: (1) control group (normal diet, 40% *w*/*v* aqueous ethanol, 60% PBS, intraperitoneal (i.p.) injections in the last 3 days); (2) DEX group (normal diet); (3) 2LDEX group, administered 2% laurel powder in feed throughout the experimental period (17 days), both before and after DEX injection. Animals in the DEX and 2LDEX groups were injected with DEX (300 μg/kg body weight/day, 40% *w*/*v* aqueous ethanol, 60% PBS, i.p.) three times at intervals of 24 h starting at day 14 to induce experimental skeletal muscle atrophy. The rats were anesthetized with isoflurane at the end of the experiment (day 17). Body weights and food intake were measured, and the lower limb skeletal muscles (tibialis anterior, gastrocnemius, soleus, and extensor digitorum longus) were removed, weighed, and flash-frozen for mRNA and protein analyses.

### 2.6. Total RNA Extraction for Real-Time PCR

Total RNA was extracted from the gastrocnemius and tibialis anterior muscles and C2C12 cells using the TRIzol reagent and RNEasy Fibrous Tissue Mini Kit (QIAGEN, Venlo, Limburg, The Netherlands). The total R+NA sample (500 ng) sample was reverse transcribed into complementary DNA (cDNA) using a PrimeScript™ RT Master Mix (Perfect Real Time; TAKARA), to perform real-time PCR analysis according to the manufacturer’s instructions. PCR was conducted on the Thermal Cycler Dice Real-Time System TP800 (TAKARA) under the following conditions: 95 °C for 30 s, followed by 40 cycles of 95 °C for 5 s and 60 °C for 30 s. The samples were matched to a standard curve generated by a 5-fold dilution of a template amplified using the same real-time PCR conditions. The RT-PCR data are presented as a fold-change and were normalized to the mRNA expression of the endogenous reference gene peptidylprolyl isomerase A (*Ppia*). The primer sequences are provided in Supplementary Table S2.

### 2.7. Western Blot Analysis

C2C12 cells or rat skeletal muscle tissue lysates (15–40 μg) were used. Electrophoresis and blotting were conducted as described previously [19]. The total protein content was measured by the Lowry assay using bovine serum albumin (BSA) as a standard. Equivalent amounts (20 μg) of protein samples were electrophoresed by sodium dodecyl sulfate-polyacrylamide gel electrophoresis and transferred onto polyvinylidene fluoride membranes. After blocking with 5% BSA in TBST for 2 h, the membranes were incubated with primary antibodies against atrogin-1/MAFbx (Abcam, Cambridge, MA, USA; Cat. #ab168372), phospho-Akt (Ser473; Cell Signaling, Danvers, MA, USA; Cat. #9271), Akt (Cell Signaling; Cat. #9272), LC3B (Cell Signaling; Cat. #2775), phospho-p70 S6 kinase (Thr389; Cell Signaling; Cat. #9234), p70 S6 kinase (Cell Signaling; Cat. #9202), phospho-4E-BP1 (Thr37/46; Cell Signaling; Cat. #2855), 4E-BP1 (Cell Signaling; Cat. #9452), and α-tubulin (Cell Signaling; Cat. #3873) at 4 °C overnight. The membranes were then washed with TBST three times and incubated with the recommended dilution of the conjugated secondary antibodies in TBST for 2 h at room temperature. The binding was visualized using an ECL Western Blotting Detection System (GE Healthcare), and the ECL signals were quantified using Ez-Capture MG (ATTO, Tokyo, Japan). Protein expression was quantitatively analyzed using the CS Analyzer 3.0 software (ATTO, Tokyo, Japan).

### 2.8. Statistical Analysis

The data are presented as mean ± standard error (SE). Statistical analysis was conducted using a one-way analysis of variance followed by Tukey’s test. For data on the effects of extracts of 34 spices and herbs on the mRNA expression of atrogin-1/MAFbx and MuRF1 and the potential cytotoxic effects of laurel on C2C12 cells, direct comparison of several groups to a single group were performed using Dunnett’s test. A *p*-value less than 0.05 (<0.05) was considered statistically significant.

## 3. Results

### 3.1. Selection of Laurel from 34 Spices and Herbs Using DEX-Induced Skeletal Muscle Cell Injury in L6 Myotubes

**Figure 1:**
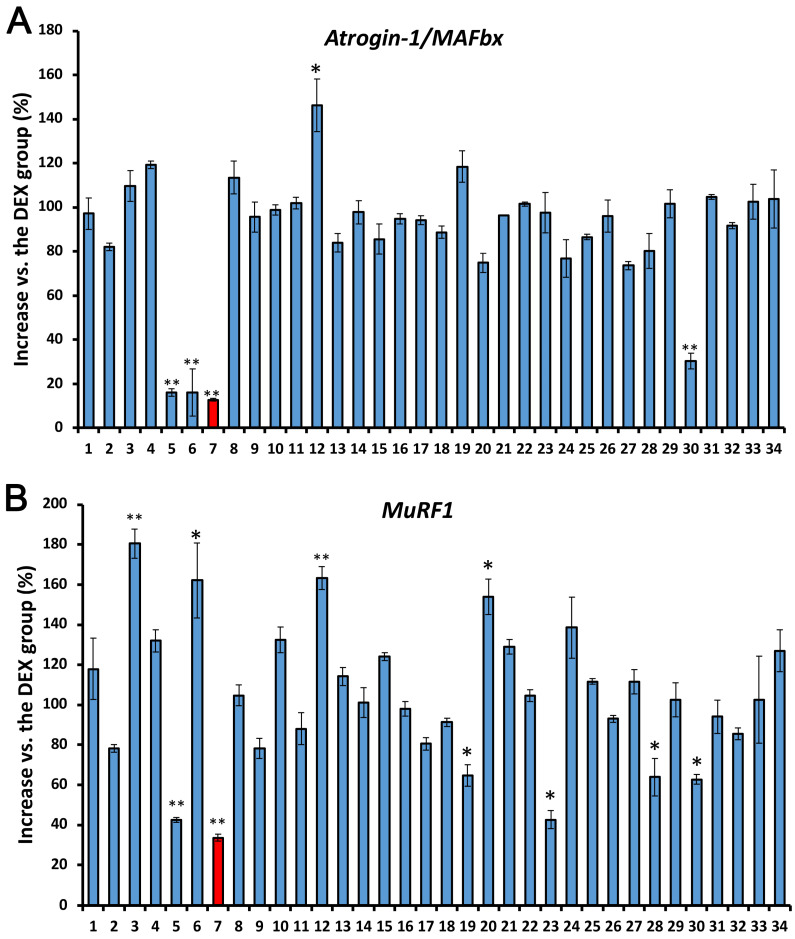
Effect of extracts of 34 spices and herbs on the mRNA expressions of (**A**) atrogin-1/MAFbx and (**B**) MuRF1 in L6 myotubes. When L6 cells reached 90% confluence, the Dulbecco’s modified Eagle’s medium (DMEM) was replaced with 2% fetal bovine serum (differentiation medium), and the cells were further incubated for 9 days. L6 myotubes were then preincubated for 12 h with different concentrations of spices and herbs. Thereafter, dexamethasone (DEX; 10 nM) was added and myotubes were incubated for 12 h. The red columns represent the laurel treatment group. Data are shown as the mean ± standard error (SE) (*n* = 4). Statistical significance was determined by Dunnett’s test. * *p* < 0.05, ** *p* < 0.01 vs. DEX group.

We determined the potential inhibitory effect of extracts of 34 spices and herbs on the mRNA expression of atrogin-1/MAFbx and MuRF1, markers of muscle atrophy [20], in L6 cells using RT-PCR. As expected, stimulation with DEX (10 nM) for 12 h increased the mRNA expression of atrogin-1/MAFbx and MuRf1 in L6 cells, whereas laurel was the most effective in suppressing the upregulation of atrogin-1/MAFbx and MuRF1 mRNA to 12.8% and 33.6%, respectively (Figure 1). Therefore, the potential effect of laurel on DEX-induced skeletal muscle atrophy was investigated in this study.

### 3.2. Laurel Suppresses the Expression of Muscle-Specific Ubiquitin Ligases in C2C12 Myotubes

**Figure 2:**
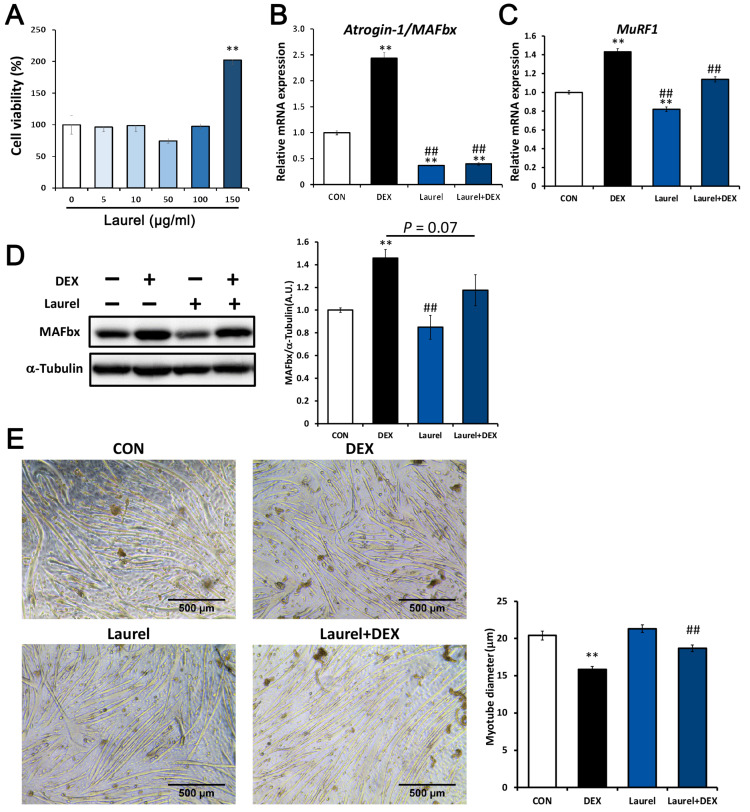
Laurel ameliorates dexamethasone (DEX)-induced C2C12 myotube atrophy by inhibiting the expression of MuRF1 and atrogin-1/MAFbx. (**A**) Effects of laurel on cell viability based on the lactate dehydrogenase (LDH) assay. (**B**,**C**) mRNA expression of atrogin-1/MAFbx and MuRF1. (**D**) Protein levels of atrogin-1/MAFbx by Western blotting; for densitometric analysis, the band intensity of atrogin-1/MAFbx was normalized to that of α-tubulin (*n* = 3). (**E**) Representative images of C2C12 myotubes after treatment with 10 nM DEX and/or laurel (50 µg/mL). A comparison of the myotube diameter in the four treatment groups is shown on the right. Data are shown as the mean ± SE (*n* = 4). ** *p* < 0.01 vs. control group. ^##^ *p* < 0.01 vs. DEX group.

The potential cytotoxicity of laurel toward C2C12 cells was determined by the LDH assay. As shown in Figure 2A, C2C12 cells were incubated with laurel for 12 h, and the cell viability was not significantly affected by the concentrations tested (5–100 μg/mL). We next investigated the changes in the levels of muscle-specific ubiquitin ligases to elucidate the effects of laurel on C2C12 cells. As depicted in Figure 2B,C, DEX significantly upregulated mRNA expression of atrogin-1/MAFbx and MuRF1 compared with that in unstimulated cells, which indicates that DEX administration enhanced the expression of these genes that are involved in proteolysis, and laurel markedly decreased their expression (by 79.5% for atrogin-1/MAFbx and16.3% for MuRF1). Notably, treatment with laurel alone reduced the expression of atrogin-1/MAFbx and MuRF1 to 36.8% and 82.0%, respectively, compared with that in the control group, reflecting its ability to reduce both the basal and DEX-induced expression of atrogin-1/MAFbx and MuRF1. Furthermore, Western blot analysis revealed that atrogin-1/MAFbx levels were significantly upregulated in DEX-treated C2C12 cells, and laurel treatment downregulated the protein levels both in the presence and absence of DEX (Figure 2D).

### 3.3. Laurel Attenuates DEX-Induced Atrophy of C2C12 Myotubes

During muscle atrophy, the activation of ubiquitinase is accompanied by a reduction in the myotube diameter. To investigate the effects of laurel on the DEX-induced reduction in myotube diameter in C2C12 cells, we examined whether laurel prevents myotube atrophy. Treatment of C2C12 myotubes with DEX significantly decreased the myotube diameter (Figure 2E). Treatment with laurel for 12 h effectively ameliorated the DEX-induced reduction in the myotube diameter. These results indicate that laurel prevents the DEX-induced increase in protein degradation and myotube atrophy.

### 3.4. Effects of Laurel on Body Weight, Food Intake, and Muscle Mass in DEX-Induced Skeletal Muscle Atrophy Model Rats

**Figure 3:**
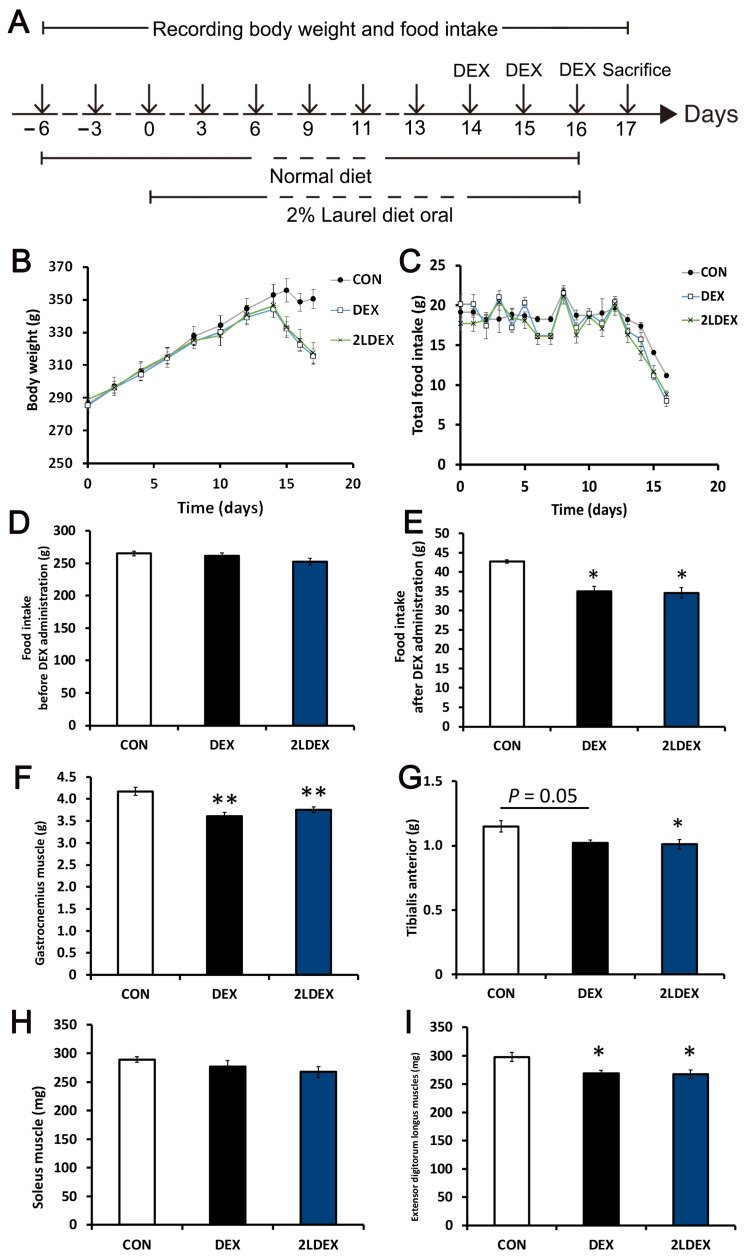
Effects of laurel on body weight, food intake, and muscle mass in DEX-induced skeletal muscle atrophy model rats. (**A**) Schematic representation of the animal experiment design. (**B**) Body weight during the experiment. (**C**) Total food intake of rats in three treatment groups. (**D**,E) The food intake of rats in three treatment groups before and after DEX administration. The weights of the gastrocnemius (**F**), tibialis anterior (**G**), soleus (**H**), and extensor digitorum longus (**I**) in three treatment groups. Data are shown as the mean ± SE (*n* = 7). Statistical significance was evaluated by Tukey’s test. * *p* < 0.05, ** *p* < 0.01 vs. control group.

A schematic representation of the animal experimental design is shown in Figure 3A. During the 2 weeks of feed treatment, all groups showed an increase in body weight (Figure 3B). However, the body weight was markedly decreased upon DEX treatment at day 14. At the end of 17 days, there was no difference in total food intake among the groups before DEX administration (Figure 3C); however, the DEX and 2LDEX groups showed lower food intake after DEX administration compared with the control group (Figure 3D,E). No differences in body weight and food intake were found between the DEX-treated groups.

In terms of organ weights at autopsy, the gastrocnemius muscle weight was significantly decreased in the DEX group compared with the control group, but there was no significant difference between the DEX-treated groups (Figure 3F). The weight of the tibialis anterior showed a decreasing trend in the DEX group (*p* = 0.05) and was significantly decreased in the 2LDEX group compared with the control group; however, there was no difference between the DEX-treated groups (Figure 3G). There was no difference in the weights of the soleus and extensor digitorum longus muscles between the DEX-treated groups (Figure 3H,I).

### 3.5. Changes in Factors Associated with Muscle Atrophy in Skeletal Muscles in Rats

**Figure 4:**
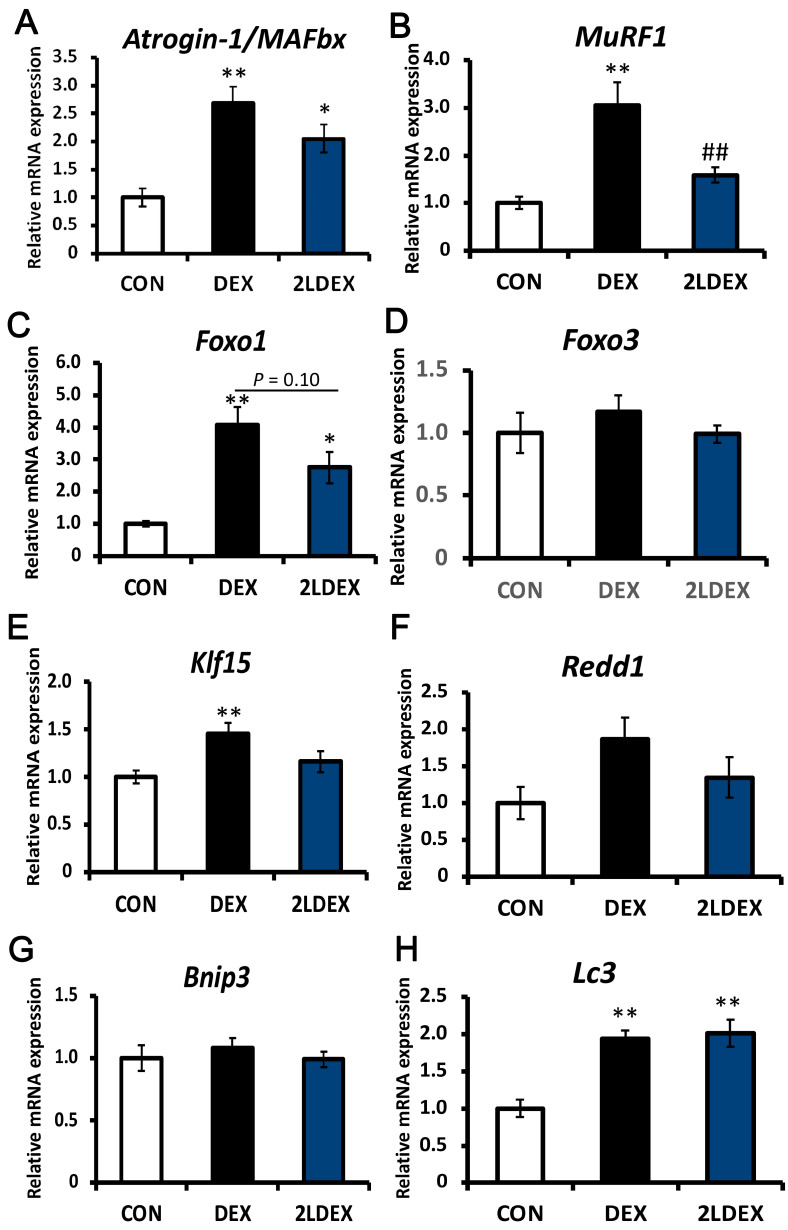
Effects of laurel on key regulatory checkpoints of the ubiquitin-proteasome and autophagolysosome systems in rat tibialis anterior muscle atrophy. mRNA levels of (**A**) atrogin-1/MAFbx, (**B**) MuRF1, (**C**) Foxo1, (**D**) Foxo3, (**E**) Kif15, (**F**) Redd1, (**G**) Bnip3, and (**H**) Lc3. Data are shown as the mean ± SE (*n* = 7). Statistical significance was determined by Tukey’s test. * *p* < 0.05, ** *p* < 0.01 vs. control group. ^##^ *p* < 0.01 vs. DEX group.

**Figure 5:**
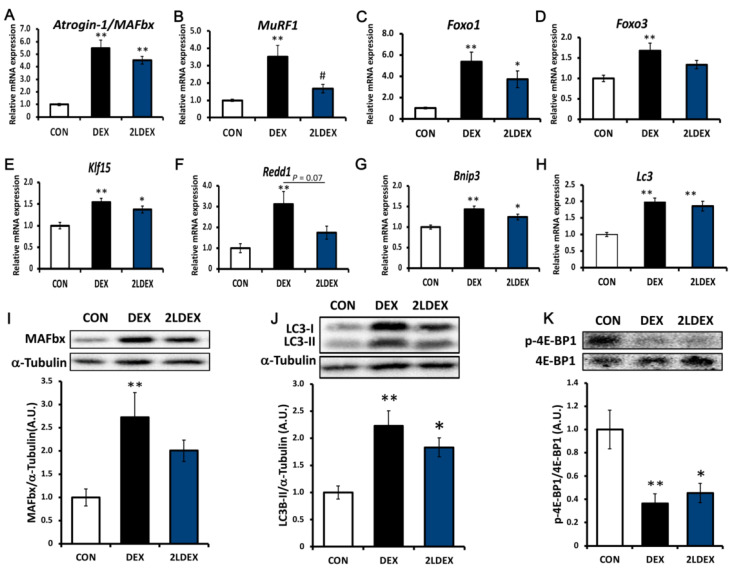
Effects of laurel on key regulatory checkpoints of the ubiquitin-proteasome system, the autophagolysosome system, and the mTOR signaling pathway in rat gastrocnemius muscle atrophy. mRNA levels of (**A**) atrogin-1/MAFbx, (**B**) MuRF1, (**C**) Foxo1, (**D**) Foxo3, (**E**) Kif15, (**F**) Redd1, (**G**) Bnip3, and (**H**) Lc3. Western blot analysis of (**I**) atrogin-1/MAFbx, (**J**) LC3, and (**K**) p-4E-BP1/4E-BP1 expression; for densitometric analysis, the band intensity of the respective protein was normalized to that of α-tubulin. Data are shown as the mean ± SE (*n* = 7). Statistical significance was evaluated by Tukey’s test. * *p* < 0.05, ** *p* < 0.01 vs. control group. ^#^ *p* < 0.05 vs. DEX group.

We further aimed to assess whether laurel has a similar effect on ubiquitin ligase in DEX-induced skeletal muscle atrophy model rats. As depicted in Figure 4A and Figure 5A,I, in the gastrocnemius and tibialis anterior tissues, the mRNA and protein expression of atrogin-1/MAFbx in the DEX group was significantly higher than those in the control group, and there was no significant difference between the DEX-treated groups. The mRNA expression of MuRF1 in the 2LDEX group was markedly decreased by approximately 47.8% and 52.0% compared with that in the DEX group in the tibialis anterior and gastrocnemius tissues, respectively (Figure 4B and Figure 5B).

The key regulatory checkpoints of ubiquitin-proteasome and autophagolysosome systems are markers of muscle atrophy. We thus explored the molecular mechanisms through which laurel inhibits skeletal muscle atrophy by detecting the expression changes of autophagy-related genes (Bnip3 and Lc3), an mTORC1 activity suppressor (Redd1), and transcription factors (Klf15, Foxo1, and Foxo3) in the tibialis anterior and gastrocnemius tissues. As illustrated in Figure 4C, in the tibialis anterior muscle, the mRNA expression of Foxo1 in the DEX group was significantly higher than that in the control group and tended to decrease in the 2LDEX group to 67.6% of the level in the DEX group (*p* = 0.10). No significant differences in the mRNA expression of Foxo3, Klf15, Redd1, Bnip3, and Lc3 were found between the DEX-treated groups (Figure 4D–H).

In the gastrocnemius muscle (Figure 5C–H), the mRNA expression of Foxo1, Foxo3, Klf15, Redd1, Bnip3, and Lc3 in the DEX group was considerably increased compared with that in the control group. The expression of Redd1 in the laurel treatment group decreased to 55.7% compared to that in the DEX group (*p* = 0.07). Collectively, these results indicate that laurel attenuates DEX-induced skeletal muscle atrophy in rats partly by suppressing the transcription of MuRF1, Foxo1, and Redd1, which are downstream factors in the ubiquitin-proteasome system, whereas DEX promotes glucocorticoid receptor (GR)-derived proteolysis. However, DEX increased the content of LC3-II at the mRNA and protein levels, while laurel had no effect, indicating that the autophagy system is probably unaffected by laurel (Figure 5I–J).

In skeletal muscle mass, REDD1 is recognized as an important negative regulator of Akt/mTOR-dependent protein synthesis that functions via Akt, 70 kDa ribosomal protein S6 kinase 1 (p70S6K1), and 4E-binding protein 1 (4E-BP1, Figure 5K). We detected the phosphorylation levels of p-Akt, p70S6K1, and 4E-BP1, downstream of mTOR. However, laurel treatment did not cause significant changes in the level of p-Akt, p70S6K1 (data not shown), and 4E-BP1 between the DEX-treated groups.

## 4. Discussion

Skeletal muscle atrophy occurs in a variety of common illnesses [21] and during aging [22] but its mechanism is not yet fully understood. Currently, atrogin-1/MAFbx and MuRF-1 have been identified as key enzymes that mediate skeletal muscle atrophy and proteolysis, and targeting their upstream cytokine expression has the potential to prevent or reverse muscle atrophy [23]. DEX, a synthetic glucocorticoid, induces muscle atrophy by regulating the expression of two muscle-specific ubiquitin ligases (atrogin-1/MAFbx and MuRF1) at the transcriptional level [20,24]. Studies on DEX-induced in vitro and in vivo models of muscle atrophy provided a good basis for this pathology and elucidated the involvement of the ubiquitin-proteasome system and autophagy [25,26,27]. Therefore, we used these models to assess the effects of laurel on skeletal muscle atrophy.

It has been reported that DEX enhances the proteolytic system and expression of genes involved in the ubiquitin-proteasome system in L6 and C2C12 cells [28]. In this study, we found that laurel significantly inhibited DEX-induced upregulation of the mRNA expression of atrogin-1/MAFbx and MuRF1 in rat skeletal muscle-derived L6 and mouse skeletal muscle-derived C2C12 cells. Laurel treatment also downregulated the DEX-induced expression of atrogin-1/MAFbx. Moreover, this had a potent inhibitory effect on the DEX-induced reduction in the myotube diameter, which is an important indicator of muscle function [29]. These in vitro findings prompted us to hypothesize that laurel might be a potential dietary therapeutic intervention to alleviate skeletal muscle cell atrophy induced by DEX.

We next investigated whether laurel treatment also improves skeletal muscle atrophy in animals and the mechanisms that regulate skeletal muscle mass. As body weight loss observed after DEX treatment is primarily attributable to skeletal muscle atrophy [30], the reduction in body weight and the associated reduction in gastrocnemius and tibialis anterior mass found in our DEX-treated rats were not incomprehensible. Gastrocnemius and tibialis anterior are skeletal muscles with relatively more fast-twitch fibers and fewer slow-twitch fibers, whereas the soleus is the opposite [31,32]. DEX has been shown to act primarily on muscles that contain type IIb fast-twitch fibers [33]. As the fiber composition of the extensor digitorum longus is similar to that of gastrocnemius [34], it is expected that the extensor digitorum longus would also suffer from similar muscle atrophy as gastrocnemius, which is consistent with our findings, that is, DEX leads to mass loss of gastrocnemius, tibialis anterior and extensor digitorum longus. In this study, muscle atrophy was observed only in gastrocnemius and tibialis anterior, which may be associated with increased expression of atrogin-1/MAFbx and MuRF1, but not in soleus, which is consistent with previous findings [33]. Although the weight of the extensor digitorum longus tended to decrease in the DEX group compared with the control group, the extensor digitorum longus is a smaller muscle that lies deeper than gastrocnemius and tibialis anterior muscles, and the difference was difficult to identify after DEX-induced muscle atrophy [31,33]. Based on the previous research and our findings, we suggest that DEX-induced fast-twitch muscle atrophy may be associated with increased expression of atrogin-1/MAFbx and MuRF1. Notably, the mRNA levels of MuRF1 were significantly reduced in animals treated with laurel compared to animals treated with DEX alone; however, laurel treatment had no effect on the mRNA expression of atrogin-1/MAFbx, as well as on the body, gastrocnemius, and tibialis anterior muscle weights.

The ubiquitin ligase MuRF1 has been shown to be directly involved in the ubiquitination and degradation of myosin during DEX treatment [35]. During this process, MuRF1 expression is directly regulated by binding to GR and indirectly via the transcription factors FoxO and KLF15. In skeletal muscles, FOXO3 is involved in regulating the expression of atrophy- and autophagy-related genes, such as Lc3 and Bnip3, thereby controlling two major pathways of protein degradation, namely the autophagic-lysosomal and ubiquitin-proteasomal pathways [36,37]. Furthermore, the protein kinase AKT is recognized as an important negative regulator of FOXO activity. Once activated, the phosphorylation of FOXO by AKT inhibits the activity of FOXO, resulting in FOXO accumulation in the cytoplasm [38]. LC3-II specifically associates with autophagosome membranes and is widely used as a standard marker of autophagosomes [39]. In our experiment, laurel did not cause changes in the levels of Akt phosphorylation, indicating that phosphorylation of the downstream target genes of FOXO was not affected by it. Together with the fact that the mRNA expression of Foxo3, Bnip3, Lc3, and LC3-II were not significantly changed by laurel, it appears that the effect of laurel on muscle atrophy is not dependent on proteolysis of the components of the autophagy system. Nevertheless, further studies are needed to unravel the pharmacokinetic potential of laurel and to assess the release patterns (gut bioavailability), biotransformation, and absorption of the bioactive form of laurel in experiments from in vitro models to in vivo environments. The results presented here should be interpreted with caution owing to differences in the pharmacodynamics of bioactive substances.

The MuRF1 promoter contains a glucocorticoid response element (GRE) and a FOXO-binding element (FBE) upstream of the transcription start site. The binding of GR and FoxO1 to the GRE and FBE, respectively, leads to synergistic activation of the MuRF1 promoter [40]. Previous studies using C2C12 myotubes demonstrated that the MuRF1 promoter binds both to GR and FOXO1 and is activated by DEX [41], which is consistent with our current findings. Mutations in either response element severely impair activation of the MuRF1 promoter. The previous findings prompted us to hypothesize that the mechanism by which laurel inhibits the mRNA expression of MuRF1 might involve inhibition of the binding of GR or FoxO1 to the GRE or FBE, respectively, in the MuRF1 promoter. In addition, luteolin and quercetin have been reported to inhibit transcriptional activity by binding to transcription factors [42,43], which suggests that the anti-skeletal muscle atrophy properties of laurel could be related to the regulation of downstream factors via the modulation of transcription factor activity. However, detailed investigations, including assessments of promoter activity using the luciferase assay and assessments of GR/FoxO binding to GRE/FBE via chromatin immunoprecipitation, are needed to better elucidate the possible molecular mechanisms.

In skeletal muscle, the nutrient-induced response of muscle protein synthesis requires mTORC1 [44,45,46]. The expression of the repressor of mTORC1 signaling, REDD1, is elevated under pathological conditions associated with muscle atrophy and is decreased under hypertrophic conditions such as pathological or mechanical exogenous causes [45]. In this study, we observed an overall tendency toward the downregulation of REDD1 expression in the 2LDEX group, probably as a result of alleviation of the suppression of mTORC1 activity, thereby promoting protein synthesis. In the mTORC1-mediated protein synthesis, the most well-defined substrates are the kinases p70S6K1 (S6K1) and eIF4E-binding protein 1 (4E-BP1), which are key regulators of protein homeostasis [47]. The activation status of mTORC1 correlates with the phosphorylation levels of S6K1 and 4E-BP1 [48]. Thus, we assessed the activity of mTORC1 by quantifying the phosphorylation levels of its downstream substrates. However, no changes in the phosphorylation levels of these substrates were observed after laurel treatment in rats. In fact, the content of REDD1 increased at 5 h after single or chronic DEX treatment but decreased to basal levels 24 h after administration [46]. In our study, the samples used were excised the day after the end of DEX administration, and as a result, REDD1 could have been degraded. To further confirm the effect of laurel on REDD1 activity and to determine whether REDD1 was degraded, it is appropriate to consider the collection of samples shortly after DEX administration.

Increasing studies have shown that laurel and laurel extracts exert many effects, including antibacterial, anti-atherosclerotic, hypolipidemic, and antioxidant properties, through different mechanisms [49,50,51]. The major components of laurel are 1, 8-cineole (up to 50%), α-terpinene, and sabinene. The major chemical components observed in essential oils mainly include monoterpenes, sesquiterpenes, and oxygenated derivatives [52]. Previous studies have revealed that the flavonoids apigenin and luteolin and the flavonol compound quercetin might have inhibitory effects on skeletal muscle atrophy [53,54,55]. Thus, it is thought that these chemical components could be the primary bioactive compounds responsible for the beneficial outcome of laurel. However, further research is warranted to reveal the potential metabolic transformation of biologically active components contained in laurel in skeletal muscle atrophy rats.

## 5. Conclusions

**Figure 6:**
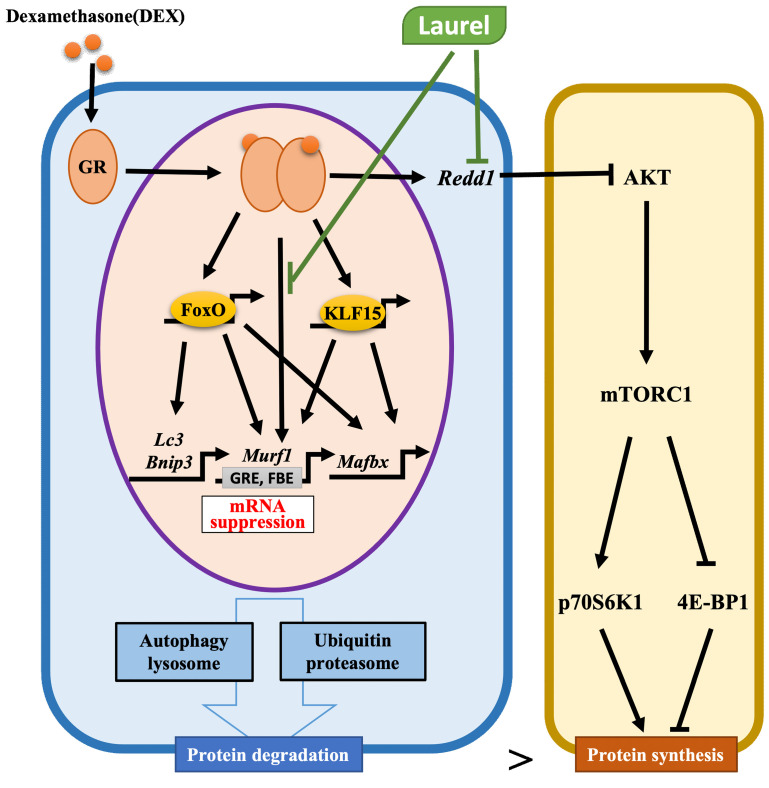
Schematic of the mechanism underlying the attenuation of DEX-induced skeletal muscle atrophy by laurel. Laurel protects against DEX-induced skeletal muscle atrophy by inhibiting the upregulation of MuRF1 mRNA, and regulating the mRNA expressions of Redd1 and Foxo1 in the muscles of rats. Moreover, inhibition of the binding of glucocorticoid receptor (GR) or FoxO1 to glucocorticoid response element (GRE) or FOXO-binding element (FBE), respectively, in the MuRF1 promoter in the skeletal muscle might be the underlying mechanism of the beneficial effects of laurel. Black arrows represent the role of DEX and green arrows represent the role of laurel.

The findings in this study demonstrate that laurel has the potential to inhibit muscle atrophy in a DEX-induced model by ameliorating the upregulation of MuRF1 mRNA and proteolysis of the components of the ubiquitin-proteasome system (Figure 6). Moreover, inhibition of GR or FoxO1 binding to the GRE or FBE, respectively, in the MuRF1 promoter of skeletal muscle might serve as a mechanism underlying the beneficial effects of laurel. These results imply that laurel could be an attractive compound for incorporation into daily meals as a seasoning to protect against skeletal muscle atrophy.

## Data Availability

The data presented in this study are available on request from the corresponding author.

## Supplementary Materials

The following supporting information can be downloaded at: https://www.mdpi.com/article/10.3390/nu14102029/s1, Table S1: Composition of experimental diets; Table S2: Primers sequences used in real-time PCR analysis. F: Forward; R: Reverse.

## References

[1] Nishikawa H., Fukunishi S., Asai A., Yokohama K., Nishiguchi S., Higuchi K. (2021). Pathophysiology and mechanisms of primary sarcopenia. Int. J. Mol. Med..

[2] Ishibashi H. (2018). Locomotive syndrome in Japan. Osteoporos Sarcopenia.

[3] Taniguchi M., Ikezoe T., Tsuboyama T., Tabara Y., Matsuda F., Ichihashi N., Nagahama Study G. (2021). Prevalence and physical characteristics of locomotive syndrome stages as classified by the new criteria 2020 in older Japanese people: Results from the Nagahama study. BMC Geriatr..

[4] Sartori R., Romanello V., Sandri M. (2021). Mechanisms of muscle atrophy and hypertrophy: Implications in health and disease. Nat. Commun..

[5] Urso M.L. (2013). Anti-inflammatory interventions and skeletal muscle injury: Benefit or detriment?. J. Appl. Physiol..

[6] Datta A., Flynn N.R., Barnette D.A., Woeltje K.F., Miller G.P., Swamidass S.J. (2021). Machine learning liver-injuring drug interactions with non-steroidal anti-inflammatory drugs (NSAIDs) from a retrospective electronic health record (EHR) cohort. PLoS Comput. Biol.

[7] Mirzoev T.M. (2020). Skeletal Muscle Recovery from Disuse Atrophy: Protein Turnover Signaling and Strategies for Accelerating Muscle Regrowth. Int. J. Mol. Sci..

[8] Budreviciute A., Damiati S., Sabir D.K., Onder K., Schuller-Goetzburg P., Plakys G., Katileviciute A., Khoja S., Kodzius R. (2020). Management and Prevention Strategies for Non-communicable Diseases (NCDs) and Their Risk Factors. Front. Public Health.

[9] English K.L., Paddon-Jones D. (2010). Protecting muscle mass and function in older adults during bed rest. Curr. Opin. Clin. Nutr. Metab. Care.

[10] Maki T., Yamamoto D., Nakanishi S., Iida K., Iguchi G., Takahashi Y., Kaji H., Chihara K., Okimura Y. (2012). Branched-chain amino acids reduce hindlimb suspension-induced muscle atrophy and protein levels of atrogin-1 and MuRF1 in rats. Nutr. Res..

[11] Shimizu N., Yoshikawa N., Ito N., Maruyama T., Suzuki Y., Takeda S., Nakae J., Tagata Y., Nishitani S., Takehana K. (2011). Crosstalk between glucocorticoid receptor and nutritional sensor mTOR in skeletal muscle. Cell Metab..

[12] Bajotto G., Sato Y., Kitaura Y., Shimomura Y. (2011). Effect of branched-chain amino acid supplementation during unloading on regulatory components of protein synthesis in atrophied soleus muscles. Eur. J. Appl. Physiol..

[13] Choudhury K., Clark J., Griffiths H.R. (2014). An almond-enriched diet increases plasma alpha-tocopherol and improves vascular function but does not affect oxidative stress markers or lipid levels. Free Radic. Res..

[14] Servais S., Letexier D., Favier R., Duchamp C., Desplanches D. (2007). Prevention of unloading-induced atrophy by vitamin E supplementation: Links between oxidative stress and soleus muscle proteolysis?. Free Radic. Biol. Med..

[15] Aoyama S., Jia H., Nakazawa K., Yamamura J., Saito K., Kato H. (2016). Dietary Genistein Prevents Denervation-Induced Muscle Atrophy in Male Rodents via Effects on Estrogen Receptor-alpha. J. Nutr..

[16] Pusceddu M.M., Hernandez-Baixauli J., Puiggros F., Arola L., Caimari A., Del Bas J.M., Baselga L. (2022). Mediterranean natural extracts improved cognitive behavior in zebrafish and healthy rats and ameliorated lps-induced cognitive impairment in a sex dependent manner. Behav. Brain Funct..

[17] Vazquez-Fresno R., Rosana A.R.R., Sajed T., Onookome-Okome T., Wishart N.A., Wishart D.S. (2019). Herbs and Spices- Biomarkers of Intake Based on Human Intervention Studies—A Systematic Review. Genes Nutr..

[18] Ito N., Ruegg U.T., Kudo A., Miyagoe-Suzuki Y., Takeda S. (2013). Activation of calcium signaling through Trpv1 by nNOS and peroxynitrite as a key trigger of skeletal muscle hypertrophy. Nat. Med..

[19] Li X., Yuan T., Chen D., Chen Y., Sun S., Wang D., Fang L., Lu Y., Du G. (2018). Cardioprotective Effects of Puerarin-V on Isoproterenol-Induced Myocardial Infarction Mice Is Associated with Regulation of PPAR-Upsilon/NF-kappaB Pathway. Molecules.

[20] Langendorf E.K., Rommens P.M., Drees P., Mattyasovszky S.G., Ritz U. (2020). Detecting the Effects of the Glucocorticoid Dexamethasone on Primary Human Skeletal Muscle Cells-Differences to the Murine Cell Line. Int. J. Mol. Sci..

[21] Yin L., Li N., Jia W., Wang N., Liang M., Yang X., Du G. (2021). Skeletal muscle atrophy: From mechanisms to treatments. Pharmacol. Res..

[22] Wilkinson D.J., Piasecki M., Atherton P.J. (2018). The age-related loss of skeletal muscle mass and function: Measurement and physiology of muscle fibre atrophy and muscle fibre loss in humans. Ageing Res. Rev..

[23] Gumucio J.P., Mendias C.L. (2013). Atrogin-1, MuRF-1, and sarcopenia. Endocrine.

[24] Gokulakrishnan G., Estrada I.J., Sosa H.A., Fiorotto M.L. (2012). In utero glucocorticoid exposure reduces fetal skeletal muscle mass in rats independent of effects on maternal nutrition. Am. J. Physiol. Regul. Integr. Comp. Physiol..

[25] Kitajima Y., Yoshioka K., Suzuki N. (2020). The ubiquitin-proteasome system in regulation of the skeletal muscle homeostasis and atrophy: From basic science to disorders. J. Physiol. Sci..

[26] Menconi M., Gonnella P., Petkova V., Lecker S., Hasselgren P.O. (2008). Dexamethasone and corticosterone induce similar, but not identical, muscle wasting responses in cultured L6 and C2C12 myotubes. J. Cell. Biochem..

[27] Hong D.H., Forsberg N.E. (1995). Effects of dexamethasone on protein degradation and protease gene expression in rat L8 myotube cultures. Mol. Cell. Endocrinol..

[28] Giron M.D., Vilchez J.D., Shreeram S., Salto R., Manzano M., Cabrera E., Campos N., Edens N.K., Rueda R., Lopez-Pedrosa J.M. (2015). beta-Hydroxy-beta-methylbutyrate (HMB) normalizes dexamethasone-induced autophagy-lysosomal pathway in skeletal muscle. PLoS ONE.

[29] Che J., Xu C., Wu Y., Jia P., Han Q., Ma Y., Wang X., Zheng Y. (2021). MiR-1290 promotes myoblast differentiation and protects against myotube atrophy via Akt/p70/FoxO3 pathway regulation. Skelet. Muscle.

[30] Fappi A., Neves J.C., Sanches L.N., Massaroto E.S.P.V., Sikusawa G.Y., Brandao T.P.C., Chadi G., Zanoteli E. (2019). Skeletal Muscle Response to Deflazacort, Dexamethasone and Methylprednisolone. Cells.

[31] Hata J., Nakashima D., Tsuji O., Fujiyoshi K., Yasutake K., Sera Y., Komaki Y., Hikishima K., Nagura T., Matsumoto M. (2019). Noninvasive technique to evaluate the muscle fiber characteristics using q-space imaging. PLoS ONE.

[32] Wens I., Dalgas U., Verboven K., Kosten L., Stevens A., Hens N., Eijnde B.O. (2015). Impact of high intensity exercise on muscle morphology in EAE rats. Physiol. Res..

[33] Krug A.L., Macedo A.G., Zago A.S., Rush J.W., Santos C.F., Amaral S.L. (2016). High-intensity resistance training attenuates dexamethasone-induced muscle atrophy. Muscle Nerve.

[34] Augusto V., Padovani C.R., Campos G.E.R. (2004). Skeletal muscule fiber types in C57BL6J. Braz. J. Morphol..

[35] Seok Y.M., Yoo J.M., Nam Y., Kim J., Kim J.S., Son J.H., Kim H.J. (2021). Mountain ginseng inhibits skeletal muscle atrophy by decreasing muscle RING fi nger protein-1 and atrogin1 through forkhead box O3 in L6 myotubes. J. Ethnopharmacol..

[36] Wiedmer P., Jung T., Castro J.P., Pomatto L.C.D., Sun P.Y., Davies K.J.A., Grune T. (2021). Sarcopenia—Molecular mechanisms and open questions. Ageing Res. Rev..

[37] Milan G., Romanello V., Pescatore F., Armani A., Paik J.H., Frasson L., Seydel A., Zhao J., Abraham R., Goldberg A.L. (2015). Regulation of autophagy and the ubiquitin-proteasome system by the FoxO transcriptional network during muscle atrophy. Nat. Commun..

[38] Dennis M.D., Coleman C.S., Berg A., Jefferson L.S., Kimball S.R. (2014). REDD1 enhances protein phosphatase 2A-mediated dephosphorylation of Akt to repress mTORC1 signaling. Sci. Signal..

[39] Runwal G., Stamatakou E., Siddiqi F.H., Puri C., Zhu Y., Rubinsztein D.C. (2019). LC3-positive structures are prominent in autophagy-deficient cells. Sci. Rep..

[40] Wang X.J., Xiao J.J., Liu L., Jiao H.C., Lin H. (2017). Excessive glucocorticoid-induced muscle MuRF1 overexpression is independent of Akt/FoXO1 pathway. Biosci. Rep..

[41] Waddell D.S., Baehr L.M., van den Brandt J., Johnsen S.A., Reichardt H.M., Furlow J.D., Bodine S.C. (2008). The glucocorticoid receptor and FOXO1 synergistically activate the skeletal muscle atrophy-associated MuRF1 gene. Am. J. Physiol. Endocrinol. Metab..

[42] Li J., Inoue J., Choi J.M., Nakamura S., Yan Z., Fushinobu S., Kamada H., Kato H., Hashidume T., Shimizu M. (2015). Identification of the Flavonoid Luteolin as a Repressor of the Transcription Factor Hepatocyte Nuclear Factor 4alpha. J. Biol. Chem..

[43] Shimizu M., Li J., Inoue J., Sato R. (2015). Quercetin represses apolipoprotein B expression by inhibiting the transcriptional activity of C/EBPbeta. PLoS ONE.

[44] D’Hulst G., Soro-Arnaiz I., Masschelein E., Veys K., Fitzgerald G., Smeuninx B., Kim S., Deldicque L., Blaauw B., Carmeliet P. (2020). PHD1 controls muscle mTORC1 in a hydroxylation-independent manner by stabilizing leucyl tRNA synthetase. Nat. Commun..

[45] Gordon B.S., Williamson D.L., Lang C.H., Jefferson L.S., Kimball S.R. (2015). Nutrient-induced stimulation of protein synthesis in mouse skeletal muscle is limited by the mTORC1 repressor REDD1. J. Nutr..

[46] Britto F.A., Begue G., Rossano B., Docquier A., Vernus B., Sar C., Ferry A., Bonnieu A., Ollendorff V., Favier F.B. (2014). REDD1 deletion prevents dexamethasone-induced skeletal muscle atrophy. Am. J. Physiol. Endocrinol. Metab..

[47] Goodman C.A. (2019). Role of mTORC1 in mechanically induced increases in translation and skeletal muscle mass. J. Appl. Physiol..

[48] Wu Z.R., Yan L., Liu Y.T., Cao L., Guo Y.H., Zhang Y., Yao H., Cai L., Shang H.B., Rui W.W. (2018). Inhibition of mTORC1 by lncRNA H19 via disrupting 4E-BP1/Raptor interaction in pituitary tumours. Nat. Commun..

[49] Stefanova G., Girova T., Gochev V., Stoyanova M., Petkova Z., Stoyanova A., Zheljazkov V.D. (2020). Comparative study on the chemical composition of laurel (*Laurus nobilis* L.) leaves from Greece and Georgia and the antibacterial activity of their essential oil. Heliyon.

[50] Jin S., Hong J.H., Jung S.H., Cho K.H. (2011). Turmeric and laurel aqueous extracts exhibit in vitro anti-atherosclerotic activity and in vivo hypolipidemic effects in a zebrafish model. J. Med. Food.

[51] Belasli A., Ben Miri Y., Aboudaou M., Ait Ouahioune L., Montanes L., Arino A., Djenane D. (2020). Antifungal, antitoxigenic, and antioxidant activities of the essential oil from laurel (*Laurus nobilis* L.): Potential use as wheat preservative. Food Sci. Nutr..

[52] Ordoudi S.A., Papapostolou M., Nenadis N., Mantzouridou F.T., Tsimidou M.Z. (2022). Bay Laurel (*Laurus nobilis* L.) Essential Oil as a Food Preservative Source: Chemistry, Quality Control, Activity Assessment, and Applications to Olive Industry Products. Foods.

[53] Kaurinovic B., Popovic M., Vlaisavljevic S. (2010). In vitro and in vivo effects of *Laurus nobilis* L. leaf extracts. Molecules.

[54] Mukai R., Nakao R., Yamamoto H., Nikawa T., Takeda E., Terao J. (2010). Quercetin prevents unloading-derived disused muscle atrophy by attenuating the induction of ubiquitin ligases in tail-suspension mice. J. Nat. Prod..

[55] Shiota C., Abe T., Kawai N., Ohno A., Teshima-Kondo S., Mori H., Terao J., Tanaka E., Nikawa T. (2015). Flavones Inhibit LPS-Induced Atrogin-1/MAFbx Expression in Mouse C2C12 Skeletal Myotubes. J. Nutr. Sci. Vitaminol..

