# Supplementary material for: Laurel Attenuates Dexamethasone-Induced Skeletal Muscle Atrophy In Vitro and in a Rat Model
Source: Nutrients. 2022 May 12;14(10):2029. doi: 10.3390/nu14102029 (PMC9143575; doi:10.3390/nu14102029)
Supplement: Supplementary file 1 [file nutrients-14-02029-s001.zip › nutrients-1706606-supplementary.pdf]

Table S1. Composition of experimental diets

Ingredient (% w/w)	AIN-93G	2% laurel-containing diet
Casein	20.00	20.00
L-cystine	0.30	0.30
β-corn starch	39.75	37.75
α-Corn starch	13.2	13.20
Sucrose	10.25	10.25
Soybean oil	7.00	7.00
Cellulose	5.00	5.00
AIN-93G Mineral mixture	3.50	3.50
AIN-93G Vitamin mixture	1.00	1.00
Laurel power	0.00	2.00

Table S2. Primers sequences used in real-time PCR analysis. F: Forward; R: Reverse.

mRNA	Species	Direction	Sequence (5′- 3′)
Mafbx	Rat	F	TTGTGCGATGTTACCCAAGAA
		R	GGTGAAAGTGAGACGGAGCA
Murf1	Rat	F	GAGGAGGAGGAGGAGGATCAA
		R	CAAGGAGCCCTAACCCCATC
Bnip3	Rat	F	CAGAGCGGGGAGGAGAAC
		R	GAAGCTGGAACGCTGCTC
Foxo1	Rat	F	CACACAGCTGGGTGTCAGGCTA
		R	GGGGTGAAGGGCATCTTT
Kif15	Rat	F	CTGCAGCAAGATGTACACCAA
		R	TCATCTGAGCGTGAAAACCTC
Lc3	Rat	F	CATGAGCGAGTTGGTCAAGA
		R	CCATGCTGTGCTGGTTCA
Redd1	Rat	F	CCAGAGAAGAGGGCCTTGA
		R	CCATCCAGGTATGAGGAGTCTT
Foxo3	Rat	F	TTCAAGGATAAGGGCGACAG
		R	GGCTGTGCAGTGACAGGTT
Ppia	Rat	F	CAAATGCTGGACCAAACACAA
		R	TCACCTTCCCAAAGACCACA
Mafbx	Mouse	F	GCTGGTGGGCAACATTAACA
		R	GTTGTAAGCACACAGGCAGGTC
Murf1	Mouse	F	CTGAGTAACTGCATCTCCATGCT
		R	TCCTTCACCTGGTGGCTATTC
Ppia	Mouse	F	GCAAATGCTGGACCAAACAC
		R	TCACCTTCCCAAAGACCACAT

Mafbx, muscle atrophy F-box; Murf1, Muscle RING finger 1; Bnip3, Bcl-2/E1B-19 kDa interacting protein 3; Foxo, Forkhead box class O; Kif15, Kinesin family member 15; Lc3, Light chain 3; Redd1, Regulated in DNA damage and development 1.
